# 2498. An evaluation of a mandatory Hepatitis C Virus screening bill in perinatally exposed infants in Kentucky

**DOI:** 10.1093/ofid/ofad500.2116

**Published:** 2023-11-27

**Authors:** Laura Guy, Lisa Yarber-Cambron, Nicholas Herring, Hunter Jennings, Katherine O’Connell, Traci Scott

**Affiliations:** Norton Healthcare, Louisville, Kentucky; Norton Healthcare, Louisville, Kentucky; Norton Healthcare, Louisville, Kentucky; Norton Healthcare, Louisville, Kentucky; Norton Healthcare, Louisville, Kentucky; Norton Healthcare, Louisville, Kentucky

## Abstract

**Background:**

In the last decade, Hepatitis C virus (HCV) rates have dramatically increased in women of child-bearing age nationally and in Kentucky. Correspondingly, there has been a documented increase in the percentage of infants born with HCV. In response to this alarming increase in HCV cases, Kentucky became the first state nationwide to implement a mandatory universal HCV screening of pregnant women in July of 2018. This study evaluates the efficacy of the mandatory universal HCV screening bill KRS 214.160 SB 250.

**Methods:**

Using laboratory data, a retrospective cohort of HCV-positive women who sought obstetric care at Norton Healthcare between January 1, 2017, and December 31, 2019, and subsequently delivered live infants were identified. Mother-infant dyads were grouped into pre- (before July 1^st^, 2018) or post-implementation (after July 1^st^, 2018) groups based on the infant’s date of birth. Manual chart reviews were utilized to identify screenings for HCV in both mothers and infants. Adequate testing was defined by the current CDC recommendations.

**Results:**

1.8% of infants in the study were diagnosed with congenital HCV infection. A modest increase of testing per CDC guidelines occurred during the post-implementation study period. Only 190 infants had at least one HCV laboratory result during the study period (68.1%). Only 55 children in the cohort were adequately tested for HCV during the study period. Fifteen infants (14.71%) born during the pre-implementation period were adequately tested per the guidelines. Only 55 (24.4%) of the infants born during the post-implementation period were tested per CDC guidelines (Fig. 1).

**Figure:**
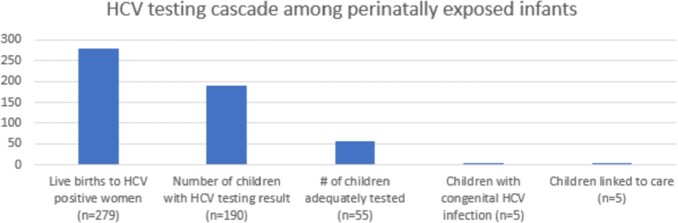

**Conclusion:**

Overall, HCV screening is still inadequate in Kentucky. However, there was a modest increase in the number of adequate HCV infant screenings. 11% of the infants in the cohort had no observable follow-up for HCV although almost all children had documented perinatal exposure to HCV. The documented vertical transmission rate in this cohort is lower than the frequently cited 5.8%. More research is required to evaluate the efficacy of the mandatory HCV screening bill in Kentucky.

**Disclosures:**

**Laura Guy, BS, CRCC**, Gilead Sciences, Inc: Grant/Research Support **Lisa Yarber-Cambron, MSN, RN, AMB-BC**, Gilead Sciences, Inc: Grant/Research Support **Nicholas Herring, BS, MPH**, Gilead Sciences, Inc: Grant/Research Support **Hunter Jennings, BS**, Gilead Sciences, Inc: Grant/Research Support **Katherine O'Connell, BS, MPH**, Gilead Sciences, Inc: Grant/Research Support **Traci Scott, MD**, Gilead Sciences, Inc: Grant/Research Support

